# A Novel Dielectric Tomography System for In-Situ Tracking Three-Dimensional Soil Water Dynamics

**DOI:** 10.3390/s18092880

**Published:** 2018-08-31

**Authors:** Song Yu, Chao Chen, Qiang Xu, Qiang Cheng, Xiaofei Yan, Zhou Yu, Yihan Ma, Haonan Chen

**Affiliations:** 1 College of Information and Electrical Engineering, China Agricultural University, Beijing 100083, China; song_y@cau.edu.cn (S.Y.); chern@cau.edu.cn (C.C.); xuqiang@cau.edu.cn (Q.X.); yz1157799015@163.com (Z.Y.); myh495836915@outlook.com (Y.M.); chenhaonan9612@163.com (H.C.); 2 School of Technology, Beijing Forestry University, Beijing 100083, China

**Keywords:** dielectric tomography, three-dimension, soil water dynamic, in-situ measurement, tube array

## Abstract

In this study, we developed a novel dielectric tomography system for in-situ tracking three-dimensional (3D) soil water dynamics. The system was designed to control a single dielectric tube sensor that automatically lowered in a PVC tube array installed in-situ to determine the water content of a soil profile, which eliminated probe-to-probe uncertainties and labor costs. Two tests for evaluating the novel system were conducted (i) to analyze and correct the positional error of the probe due to out-of-step errors of stepper motors, and (ii) to track and visualize 3D soil water temporal variations in a soil tank with heterogenetic bulk densities and initial water contents under drip irrigation. The results show that the positioning correcting algorithm combined with starting point alignment can minimize the positioning error of the probe during the 3D tomography. The single drip emitter test illustrated spatial and temporal variations of soil water content due to heterogeneous soil properties in vertical and horizontal directions around the access tube array. Based on these data, 3D distributions of soil water dynamics were visualized. The developed tomography system has potential application to be extended to the local scale in a greenhouse or the large scale in an agricultural field. Future research should explore the performance for agricultural crop irrigation or for modeling and validating soil water flow or hydrological process under either steady state or non-steady state condition.

## 1. Introduction

Monitoring variation of soil water content (SWC) in three dimensions (3D) can benefit researchers studying agricultural water management, soil hydrological process, fluid pollutant transport phenomena, etc., in heterogeneous soils [1] where spatial variability of soil properties and field and meteorological conditions significantly affect the distribution of SWC at different scales [2,3,4,5,6,7,8]. With this intention, diverse sensor techniques such as time-domain reflectrometry (TDR) [9], frequency-domain impedance (FDI) sensor [8], neutron moisture meter (NMM) [10], heat pulse (HP) sensor [11] and electrical resistivity tomography (ERT) [12] have been developed and widely used to monitor the temporal variations of soil water content at multiple positions.

In early studies, needle-structure-based sensors (e.g., TDR, FDI, HP, etc.) with multiple probes were used to automatically monitor 3D soil water dynamics [9]. However, the installation of the multiple probes has to excavate a large pit to insert a series of probes at different depths and sites, inevitably disturbing the soil structure surrounding the sensors [10,13,14]. Besides, multiple probes tended to be costly and accuracy prohibitive due to probe-to-probe uncertainties and the site-specific calibrations for each probe increased labor cost, which made this implement impractical for most researchers. Tube-type sensors (e.g., NMM, tube-TDR, tube-FDI) can in-situ determine 3D spatial and temporal variations of SWC with one probe moving in multiple access tubes without altering the soil structure. However, so far there is no instrument that can automatically measure the variations of SWC without human labor, which is a disadvantage compared with the implement of multiple probes. Although ERT is a non-destructive method and can be used for automatically determining the variations of SWC in 3D [12,15], there are many factors (e.g., salinity, temperature, systematic errors due to poor electrode contact, etc.) influencing the soil electrical resistivity [16], and the basic principle of the data interpretations of ERT has to link the electrical measurements with the soil characteristics and function, which is more complicated than that of NMM, TDR or FDI. In recent years, neutron radiography and tomography (NT), X-ray computed tomography (CT) and magnetic resonance imaging (MRI) have shown great potential as they provide high spatial and temporal resolutions to measure SWC at small distances [7]. However, these new techniques have shown poor signal to-noise ratio for MRI, insensitivity to water for X-ray CT, harmful radiation to human body for NT and higher costs, and they are not suitable for field measurement.

This study attempts to develop a novel dielectric tomography system for in-situ monitoring 3D soil water dynamics. The system was designed to use a single FDI probe automatically moving in three mutually orthogonal directions and lowering in across PVC tube array to eliminate probe-to-probe uncertainties. Two tests for evaluating the novel system were conducted (i) to analyze and correct the positional error of the probe due to out-of-step errors of stepper motors, and (ii) to track and visualize 3D soil water temporal variations in a soil tank with heterogenetic bulk densities and initial water contents under drip irrigation.

## 2. Materials and Methods 

### 2.1. Description of Dielectric Tomography System

**Figure 1:**
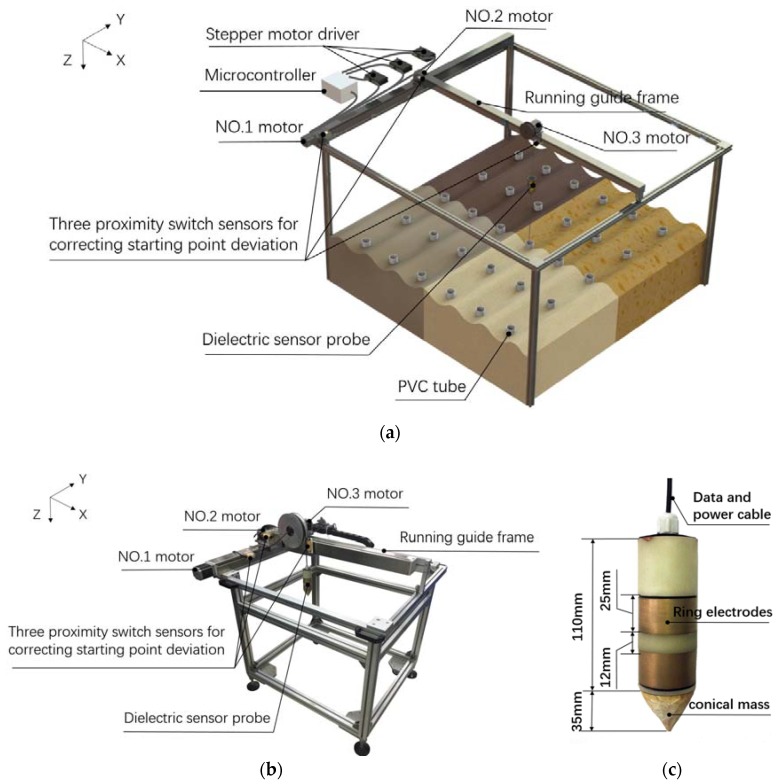
(**a**) Framework diagram of the three-dimensional tomography system; (**b**) photograph of the system and (**c**) the dielectric probe.

The framework of the three-dimensional tomography system was made of aluminum alloy, as shown in Figure 1. The size of the framework can be adjusted according to area of a specific experimental plot. There are three stepper motors (57HS7630A4, SUMTOR Electric Equipment Co., Ltd., Wuxi, China) and three proximity switch sensors (E18-D50NK, PeiYi Electric Co., Ltd, Wenzhou, China) mounted on the framework. The No. 1 motor controlled running guide frame moving in horizontal Y-Axis direction, No. 2 motors controlled dielectric sensor probe together with No. 3 motor moving in horizontal X-Axis direction perpendicular to Y-Axis and the No. 3 motor controlled the dielectric probe moving in vertical Z-Axis direction. The three proximity switch sensors were used for correcting the starting point deviation of the three mutually orthogonal directions. A PVC-tube array was installed in-situ under the framework without disturbing the soil structure [14,17].

The shape of the dielectric probe was designed (Figure 1c) to be cylindrical to match the size of the PVC tube. The thickness of the PVC tube wall is 3.5 mm. The inner diameter (i.d.) of PVC pipe is 48 mm, 1 mm larger than the sensor diameter (47 mm, outer diameter, o.d.), ensuring that the probe can move smoothly in the PVC tube and minimize the air gap induced measurement error [18]. According to the results reported by Sun et al. (2014) [8], the sensor’s area of sensitivity suggested a radius of 5.5 cm from the PVC tube wall. A conical mass was attached to the lower part of the probe to keep it vertical and facilitate the probe lowering in the tube. The output signal cable extended from the top of the probe and connected with microcontroller through a kinking wheel, which is dragged by No. 3 motor. The top of the probe was wrapped with black paper to help the proximity switch sensors to correct vertical position of the probe.

**Figure 2:**
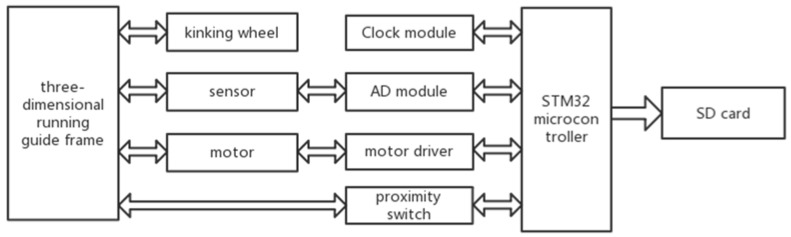
Diagram of the system design.

Microcontroller STM32 (STMicroelectronics, STM32F429) was used to control motors and collect the data from the dielectric sensor and the proximity switch sensors (Figure 2). The timer in the STM32 could generate PWM wave that controls the motor moving a pre-set distance relating to the number of PWM wave. The microcontroller was connected to the dielectric sensor probe, supplying power to the dielectric sensor probe and collecting the SWC data. The data was stored in a storage module with an 8 GB SD-card loaded on the STM32.

### 2.2. Sensor Principle and Calibration

The dielectric sensor used in this study was designed and implemented based on frequency domain method, of which the concept was first introduced by [17]. Dielectric sensors can indirectly determine volumetric soil water content (VSWC) because the relative permittivity of water (ε_water_/ε_0_ = 81, 20 °C) is much higher than those of soil minerals (ε_soil_/ε_0_ = 3) and air (ε_air_/ε_0_ = 1). The sensor probe generates an external electric field through two ring electrodes, and the variations of soil permittivity cause changes of probe impedance, as shown in Figure 1c. The soil water content can be obtained by measuring the impedance of the sensor probe.

(1) $$Z_{P}\left(\varepsilon \right)=\frac{U_{b}}{U_{a}-U_{b}}Z_{0}$$

where *Z_P_* is the electrical impedance of probe; *ε* is the relative permittivity of wet soil; *U_a_* and *U_b_* are the outputs of two wave detectors; *Z*_0_ is the balance impedance. The operating frequency (*f*) in sensor circuit is set at 100 MHz because some previous studies [17,19,20] emphasized that the measured VSWC could be made at high frequencies usually higher than 30 MHz to minimize the contribution of the soil electrical conductivity. Sun et al. (2012; 2014) [8,14] detailed descriptions of the dielectric tube sensor used in this study.

For the sensor calibration, the soil samples were collected from an experimental field in China Agricultural University, then passed through a 2-mm mesh screen and were oven-dried at 105 °C for 48 h. After the samples were remoistened to different moisture levels from dry to near saturation, they were sealed in PVC cylinders (i.d. 30 cm, height 30 cm) where a segment of the PVC access tube stood erect at the center of each cylinder.

### 2.3. Correcting Algorithm for Minimizing Out-of-Step Errors of Three Stepper Motors

No. 1 and No. 2 motors control the frame moving in X and Y directions and the pitch of the lead screw that acts as a transmission in the guide frame is 5 mm. That is, the motor rotates one cycle and the guide frame theoretically moves 5 mm. The No. 3 motor controls the kinking wheel rotating. The motor rotates one circle and the sensor moves a circumference of the kinking wheel. The number of pulses required to rotate one step subdivision is calculated as:

(2) $$N_{f}=\frac{360^{{}^{\circ}}}{\theta_{e}}\times n$$

where *n* is the subdivision factor, $\theta_{e}$ is the step angle, and $N_{f}$ is the number of pulses required for the stepper motor to rotate one step subdivision. All of the stepping angles of the three motors used were 1.8°, and the three stepper motor drivers (24 V, BH-MSD4805) were set to 100 subdivisions. Thus, each motor theoretically needed 10,000 pulses to make a circle. The advancement of the guide frame in the X and Y directions by 10 mm requires a pulse of 20,000 steps. In the Z direction, the number of pulses (*N_z_*) to rotate a certain distance d (in mm) (i.e., the measurement increment) is:

(3) $$N_{Z}=\frac{d}{L}\times 10,000$$

where *L* is the circumference of the kinking wheel. It is known that the diameter of the kinking wheel is 145 mm, and thus for every *d* = 50 mm rotation of the kinking wheel, No. 3 motor theoretically requires 1098 steps. Supposed that the number of steps required to theoretically move a certain distance d is M, and the actual moving distance measured is *d’*, a compensation index (CI) for minimizing the out-of-step errors of the stepper motors is presented

(4) $$\mathrm{CI}=\frac{d}{d^{\prime }}$$

and the corrected number of steps (M’) required to actually move the distance *d* is presented as follow:

(5) $$M^{\prime }=M\times \mathrm{CI}$$

**Figure 3:**
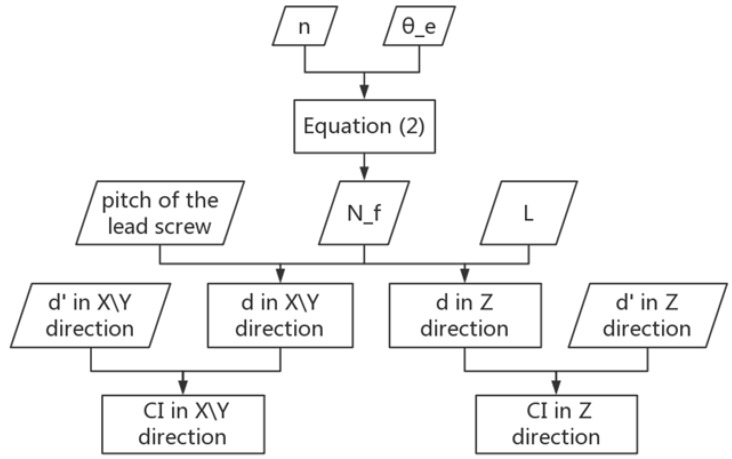
Flowchart of the algorithm for minimizing out-of-step errors of three stepper motors.

The flowchart of the algorithm for minimizing out-of-step errors of three stepper motors was shown in Figure 3.

### 2.4. Experimental Procedure

**Figure 4:**
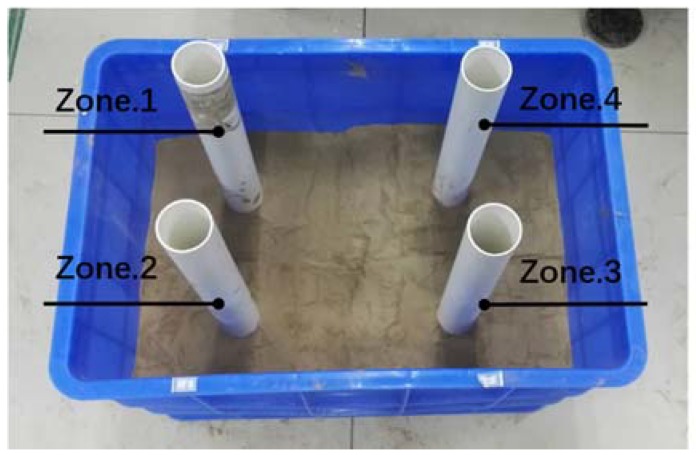
A soil tank with a four-tube array installed in-situ.

The experiment was carried out in a soil tank under laboratory conditions. The size of the tank is 555-mm length × 355-mm width × 430-mm height, as shown in Figure 4. To test the developed tomography system for 3D SWC measurements, we created a heterogeneous soil condition. The air-dried soil samples were separated into two groups and moistened at two different water content levels for preparing layered soil condition (Layer-1 and Layer-2). We randomly collected and oven-dried three samples of each group to determine initial gravimetric SWC. The initial gravimetric SWC in Layer-1 was 0.130 ± 0.003 g g^−1^ and that in Layer-2 was 0.095 ± 0.003 g g^−1^. A 20-cm height soil sample (Layer-1) filled in the tank was divided into four parts with identical volume but different bulk densities (Zone-1: 1.428 g cm^−3^; Zone-2: 1.165 g cm^−3^; Zone-3: 0.902 g cm^−3^; Zone-4: 0.729 g cm^−3^). Prior to this, a 10-cm height soil sample (Layer-2) was firstly filled at the bottom of the soil tank with identical bulk density (1.10 g cm^−3^). The detailed information was shown in Table 1. In this way, the spatial variability of soil properties (bulk density and initial SWC) at horizontal and vertical profiles was achieved. The PVC tube with a height of 560 mm was installed in-situ at the center of each zone to measure SWC.

A single-dripper (dripping rate: 0.791 L/h) was put in the central part of the soil tank, keeping the same distance from each tube to ensure identical irrigation rate of soil water movement at both vertical and horizontal directions for the four zones. Monitoring cycle of the system was set to five minutes with the measurements of the four PVC tubes totally lasting two minutes. The rotation velocity of the three motors can be adjusted according to the plot scale to be measured and monitoring cycle. In one measurement, No. 1 and No. 2 motors guided the sensor probe to the top of a specific PVC tube as precise as possible, and then No. 3 motor turned clockwise to lower the sensor probe along the Z-Axis in the PVC tube at a pre-set maximum depth and subsequently turned counterclockwise to advance the probe in 50 mm increments from bottom to top and simultaneously measured the SWC. The whole measurement needs about 144 s, among them, No. 1 runs once for 9 s, No. 2 runs for 13.5 s, No. 3 drops for 8.7 s, and rises for 16 s. For a larger scale measurement, the measurement period will be longer. After finishing the measurements of four PVC tubes, the sensor returned to the initial position based on the signals from the three proximity switch sensors. The three-dimensional dynamics of SWC were obtained and presented by the interpolation function with Matlab software (MATLAB 7.0, MathWorks, Natick, MA, America).

## 3. Results and Discussion

### 3.1. Dielectric Sensor Calibration and Test of Acquiring the Correcting CIs for the Out-of-Step Correction

**Figure 5:**
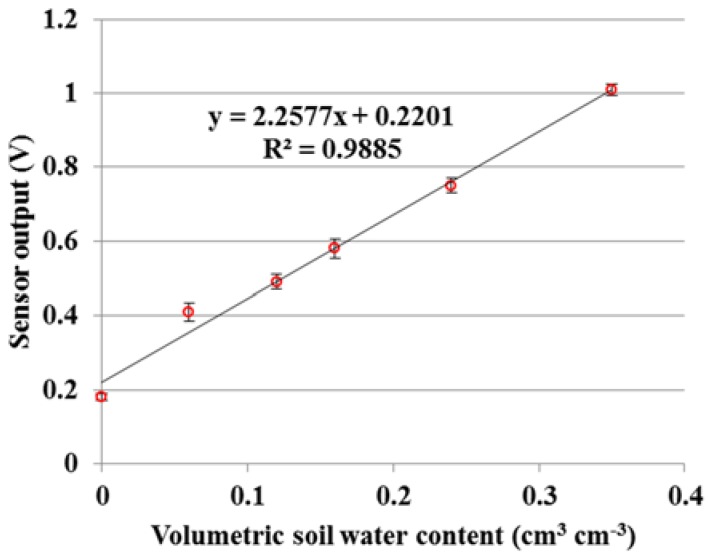
The relationship between volumetric soil water content (VSWC) and the output of the dielectric sensor.

The dielectric sensor was calibrated with a loamy soil (sand: 42.1%, silt, 44.2%, clay: 13.7%) under laboratory condition at a room temperature of 21 ± 1 °C. The level of VSWC ranges from 0 to 0.35 cm^3^ cm^−3^ as shown in Figure 5. The calibration curve obtained is linear expression y = 2.2577x + 0.2201 with a high determination coefficient (R^2^) equaling to 0.9885 between the VSWC and sensor output voltage.

The test results of the acquired CIs for correcting the out-of-step errors of the three stepper motors are shown in Table 2. For each 10 mm advancement of the guide frame driven by No. 1 and No. 2 motors, it is theoretically necessary to use 20,000 pulses, but actually requires 24,840 pulses, with the CI equaling to 1.242. It theoretically requires 1098 pulses for the No. 3 motor to drive the kinking wheel rotating 50 mm increment, but actually requires 1166 pulses, with the CI equaling to 1.062.

### 3.2. The Temporal Variations of SWC along the Vertical Direction (Z-Axis)

**Figure 6:**
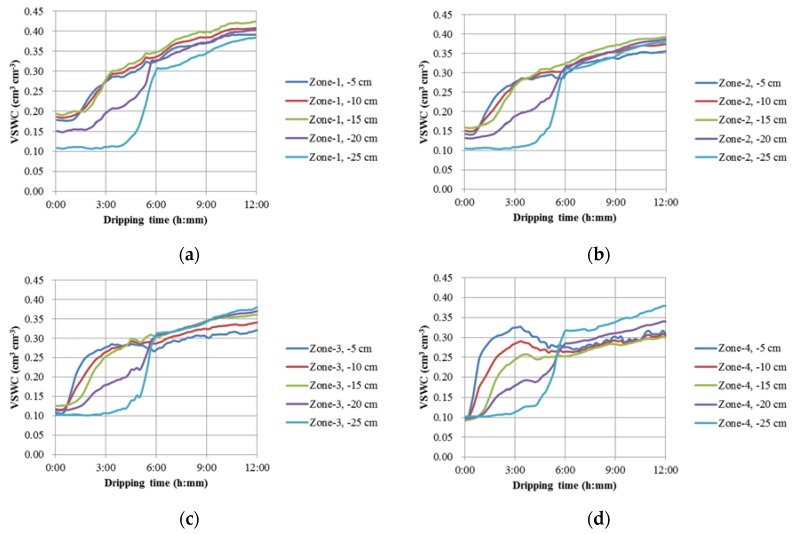
Temporal variations of the soil water content along the four tubes (Z-Axis) ((**a**) Zone-1, (**b**) Zone-2, (**c**) Zone-3 and (**d**) Zone-4) under single-drip irrigation condition.

Figure 6 shows the temporal variations of the soil water content along the four tubes (Zone-1, Zone-2, Zone-3 and Zone-4) under single-drip irrigation condition. From these curves, it can be generally seen that (i) due to the identical initial gravimetric SWC but different soil bulk densities for the four zones, the initial VSWC at Zone-1 (circa (ca.) 0.186 cm^3^ cm^−3^) was highest and that at Zone-4 (ca. 0.095 cm^3^ cm^−3^) was lowest as expected; (ii) the water gradually moved in horizontal direction and simultaneously infiltrated into soils from the depth of 5 cm (−5 cm) to that of 15 cm (−15 cm) with first increases of the VSWC values at −5 cm, followed by the increases of the VSWC values at −10 cm and then those at −15 cm at Layer-1; take Zone-1 for instance, the time that the VSWC value started to increase at −5 cm was around 1:00, then that at −10 cm was around 1:20, and finally that at −15 cm was around 1:50; (iii) a transitional zone existed at the depth of 20 cm (−20 cm) where the volume of sensitivity of the dielectric sensor includes the soil water at both Layer-1 and Layer-2; (iv) at Layer-2 (−25 cm), the initial VSWC values at Zones-1, -2 and -3 were lower than those at Layer-1; however, at Zone-4, the initial VSWC values at Layer-2 were close to those at Layer-1 due to the lower density at Layer-1 of Zone-4. These data shows that the developed tomography system for vertical measurement could contribute significant information to improving understanding of the soil water infiltration and redistribution.

### 3.3. The Temporal Variations of SWC along the Horizontal Direction (X- and Y-Axis)

**Figure 7:**
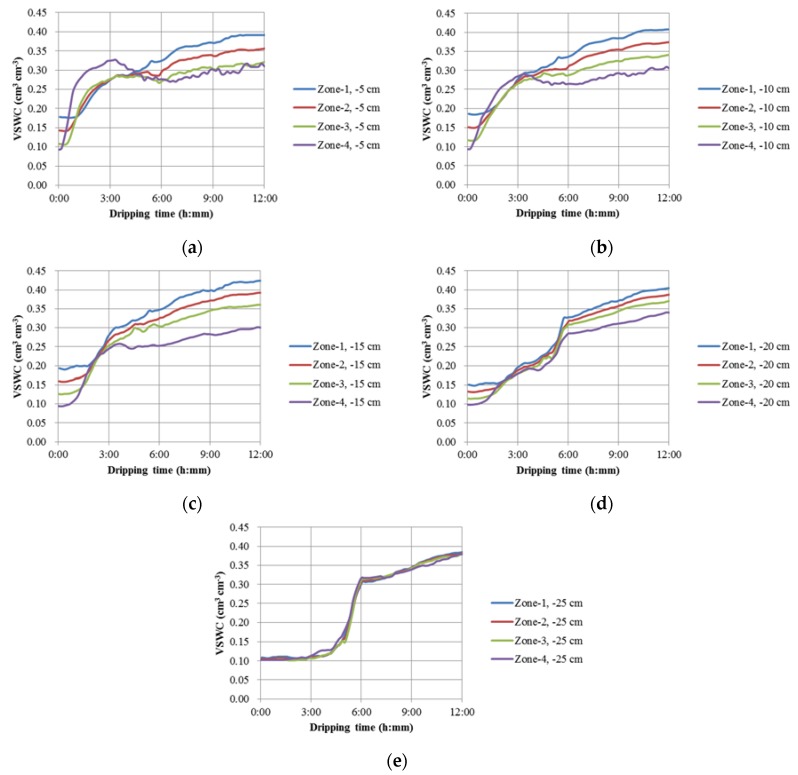
Temporal variations of the soil water content along the horizontal direction (X- and Y-Axis) at the depths of (**a**) 5 cm (−5 cm), (**b**) 10 cm (−10 cm), (**c**) 15 cm (−15 cm), (**d**) 20 cm (−20 cm) and (**e**) 25 cm (−25 cm) under single-drip irrigation conditions.

Figure 7 shows the temporal variations of the soil water content along the horizontal direction (X- and Y-Axis) at the depths of 5 cm (−5 cm), 10 cm (−10 cm), 15 cm (−15 cm), 20 cm (−20 cm) and 25 cm (−25 cm) under single-drip irrigation condition, which clearly reflected the heterogeneous soil conditions with different bulk densities. For example, in Figure 7a, due to the highest soil bulk density in Zone-1, the VSWC values did not increase until ca. 1 h later, which indicated the water infiltration rate were lowest compared with the other three zones (Zone-2, ca. 40 min; Zone-3, ca. 30 min; Zone-4, ca. 20 min). However, the differences of the infiltration rates gradually disappeared at Layer-2, indicating a longer delay of the water infiltration between Layer-1 and Layer-2, especially when the soil bulk density was lower. This may attribute to the higher soil porosity that led the water to move faster in this zone and thus, the soil matric potential was increased to a higher value in comparison with the soils with lower porosity, and the water may easily move from the zones with lower soil density to those with higher soil bulk density.

### 3.4. Tomography of 3D SWC Distribution under Drip Irrigation Condition

**Figure 8:**
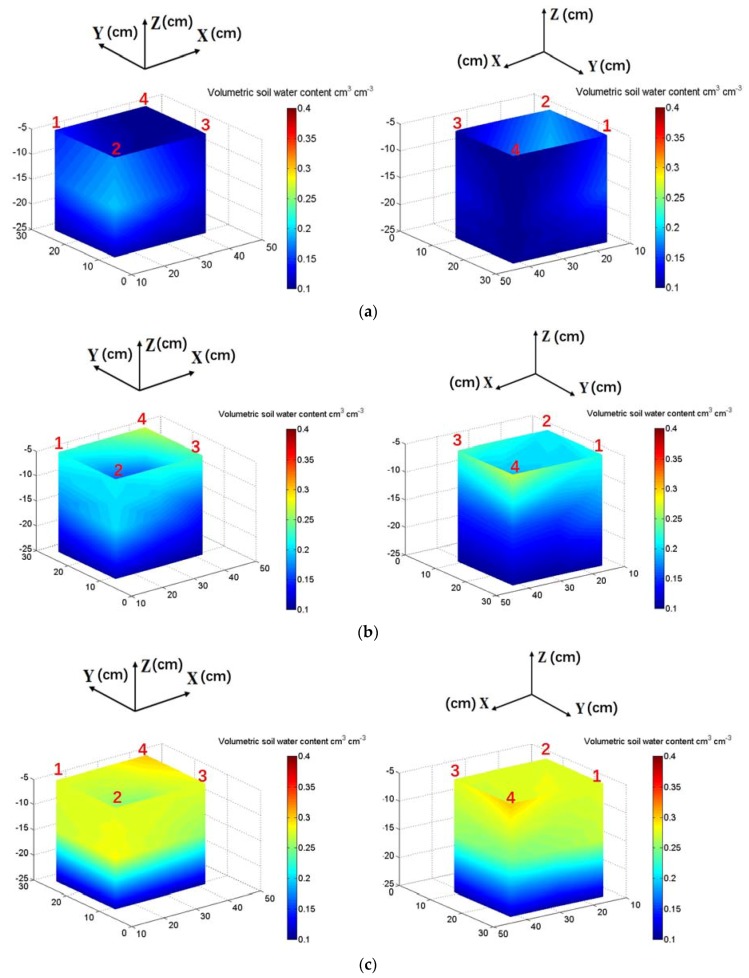

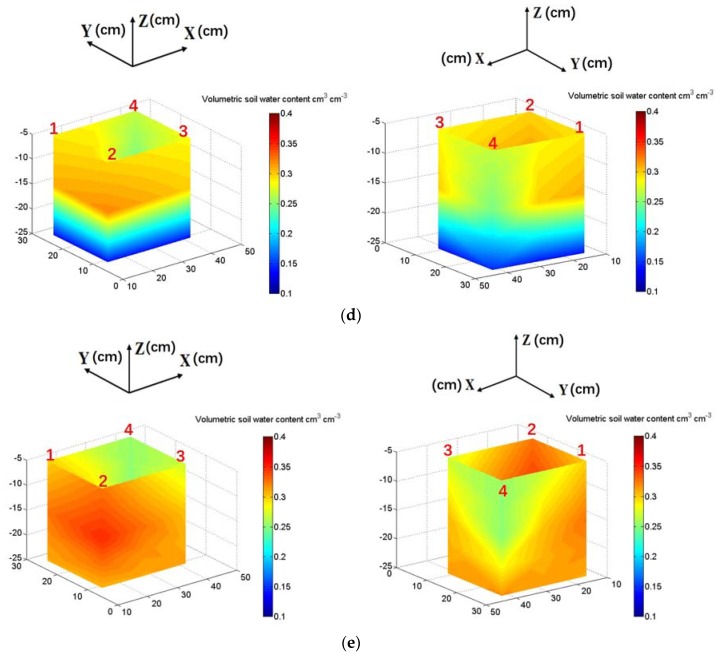
Three-dimensional temporal pattern of VSWC observed by the dielectric tomography system running under single-dripper irrigation condition (**a**) 3D SWC map at starting time (0:00); (**b**) 3D SWC map at 1.5 h (1:30); (**c**) 3D SWC map at 3 h (3:00); (**d**) 3D SWC map at 4.5 h (4:30); (**e**) 3D SWC map at 6 h (6:00).

Figure 8 exhibits a group of 3D SWC maps generated from the data in Figure 6 and Figure 7 at the time of 0:00, 1:30, 3:00, 4:30 and 6:00. At the time of 0:00, the 3D distribution map of the SWC (Figure 8a) at Layer-1 (−5, −10 and −15 cm) shows a higher initial VSWC at Zone-1 with a lighter blue color. At the time of 1:30 (Figure 8b), due to the fast infiltration at Zone-3 and Zone-4, the color at −5 cm turned to green, with the VSWC values higher in comparison with those at Zone-1 and Zone-2. At the time of 3:00 (Figure 8c), the VSWCs were saturated at −5 cm and the wet front penetrated deeper to the −15 cm. At the time of 4:30 (Figure 8d), the VSWCs at −5, −10 and −15 cm were saturated and the wet front had already arrived at the −20 cm. At the time of 6:00 (Figure 8e), the wet front penetrated over the depth of 25 cm and the soil in the tank were almost saturated.

## 4. Conclusions

This study successfully presented a novel dielectric tomography system designed to control a single dielectric probe automatically moving in three mutually orthogonal directions across multiple tubes. The series of tests demonstrate the technical performance of the dielectric tomography system for in-situ tracking 3D soil water content variations, which is unavailable previously. The developed tomography system has potential application to be extended to the local scale in a greenhouse. Future research should explore the performance for agricultural crop irrigation or for modeling and validating soil water flow or hydrological process under either steady state or non-steady state conditions.

## Figures and Tables

**Table 1:** Soil bulk densities preparation for Layer-1 and Layer-2 at Zones-1, -2, -3 and -4.

	Soil Bulk Densities (g cm^−3^)	
	Layer-1 (−20~0 cm)	Layer-2 (−30~−20 cm)
Zone-1	1.428	1.10
Zone-2	1.165	1.10
Zone-3	0.902	1.10
Zone-4	0.729	1.10

**Table 2:** Results of the acquired compensation indices (CIs) of the three stepper motors.

	No. 1 Motor	No. 2 Motor	No. 3 Motor
compensation index (CI)	1.242	1.242	1.062
